# Matured Manure and Compost from the Organic Fraction of Solid Waste Digestate Application in Intensive Apple Orchards

**DOI:** 10.3390/ijerph192315512

**Published:** 2022-11-23

**Authors:** Daniela Bona, Andrea Cristoforetti, Roberto Zanzotti, Daniela Bertoldi, Nicole Dellai, Silvia Silvestri

**Affiliations:** Fondazione Edmund Mach, Via E. Mach, 1, 38010 San Michele All’adige, Italy

**Keywords:** compost from OFMSW digestate, matured manure, apple orchards, amendment, fertilizers

## Abstract

In intensive fruit growing systems, the recovery and maintenance of soil fertility play a crucial role in both environmental protection and sustainable support to plant productivity. The circular economy approach adopted at the EU level strongly promotes the use of organic products instead of mineral fertilizers. This work focuses on two different soil improvers, compost from the organic fraction of municipal solid waste digestate (CO) and “matured” manure, produced after a fast and controlled aerobic treatment in an aerated pile (MM), which were applied in three apple orchards with different soil tillage. The soil improvers have been characterized for amendment and fertilizing properties. After the amendment, the soils were sampled twice a year (Spring and Autumn) for three years. Each sample has been characterized for texture, pH, cation exchange capacity, nutrients, soil organic matter, and micronutrients. The amendments obtained differed on C, N, P, and K contents, but had similar biological stability. The main effects on soils were the increasing of N and soil organic matter after compost application, while the use of matured manure mainly act on available P and exchangeable K. The treatments showed significant effects among fields with a linear increasing trend only for compost. Matured manure showed more effects in earlier times. The data collected aim to improve the knowledge about sustainable management of soil organic matter and organic nutrients in intensive fruit-growing agriculture by using local products.

## 1. Introduction

In the last years, the intensification of agriculture has led to an increase in the use of chemical fertilizers to sustain crop yield and meets the demands of food and feed for the growth of the world population. This results in soil degradation, with loss of organic matter, greenhouse gas emissions increasing erosion, contamination, acidification, salinization, and loss of biodiversity [1]. In addition to food production, soils provide a broad range of other environmental and cultural ecosystem services, such as carbon storage and greenhouse gas (GHG) thus essential for humanity [2]. Soil organic matter (SOM) plays a crucial role in sustaining the physical, chemical, and biological fertility of the soil. Agricultural production results in the mineralization of this organic matter content determining over the long term a reduction of organic carbon stock [1]. The less use of agrochemicals and fertilizers in favur of recovered nutrients together with the maintenance of soil fertility by rebuilding and managing the SOM in agricultural lands [3,4] will permit the farmers to restore healthy soil and to support more sustainable crop production systems.

The recent EU goals (Farm to Fork strategy) aim to reduce the use of chemical pesticides (50%), fertilizers (20%), and nutrient losses (at least 50%) by 2030. Achieving sustainable soil management in the EU is a crucial action contributing to the 2030 Agenda and Sustainable Developments Goals [5]. At the same time, the recent policies lead to better and more efficient use of nutrients by exploiting waste and biomass at the local level, as expected in the Action Plan for Circular Economy and the Bioeconomy Strategy [6,7]. Moreover, the recent European Regulation on Fertilizers provides the limits and characteristics of the amendment from biomass and waste [8]. The recent EU goals expect that bio-waste will replace up to 30% of the inorganic fertilizers currently used [9].

Livestock is historically the main nutrient provider for sustaining crop growth [9,10]. A study by Case et al. (2017) concerning the use of bio-based fertilizers by farmers showed that the main issue regarding the use of organic products instead of chemical fertilizers was the uncertainty of nutrient content and the difficulty in planning its use [11]. Therefore, it is very important to work on both the production of suitable amendments and the study of the impact on mineral fertilizers reductions, mainly in monocultures systems, i.e., orchards or vineyards [12]. The manure composition depends on several factors such as the feedstock used (animal, vegetable, or mixed), the season, and the management of the litter [13]. Many technologies and treatments are available to obtain good products from manure, such as anaerobic digestion, composting, and thermochemical treatments. In this work, the first product considered, namely matured manure (MM), was obtained after a faster process of aeration and maturation of manure. The controlled maturation process is simpler than composting and aims to overcome the costs and operational needs of composting, finding a product more suitable for the orchard application. The process led to obtaining a product more stable, with higher content of organic nitrogen and with a loss of water content, similar to compost. The physicochemical properties of manure support its use as a soil improver and organic fertilizer, and a recent study showed that manure quality is more important than manure quantity [14].

The compost (CO) application in apple orchards has positive effects on both soil quality and plant productivity [15]. The CO can be obtained from several feedstocks, including the organic fraction of municipal solid waste (OFMSW). The introduction of anaerobic digestion (AD) technologies for the conversion of food waste into bioenergy led to the production of large quantities of digestate, an organic by-product that contains partially degraded material, microbial biomass, nutrients, and minerals [16,17]. Digestate stabilization through composting provides a sustainable option to produce nutrient-rich and pathogen-free organic fertilizers while reducing storage volume, completing the nutrient cycle loop, and reaching a very profitable rural-urban synergy [16,18]. The recent studies focused on OFMSW digestate composting highlighted some process difficulties in attaining the thermophilic phase, ammonia emission, and low quality of the compost [19,20].

The two processes lead to products with a different amendment and fertilizing properties, but they are currently being used in the same manner in managing the SOM in apple orchards. The knowledge of the main and specific effects on soil, considering both soil organic matter content and nutrient dynamics is very important to reach a better use in intensive agriculture through efficient fertilization strategy and to avoid pollution phenomena (loss of nutrients and GHG emissions) due to surplus of nutrients, which would be difficult to relocatable. Moreover, the recovery of local nutrients could contribute to creating a positive synergy among sectors, particularly in the mountain region, where often, livestock, fruit growing, and urban area coexist. 

The aims of this work were (a) to define the quality of amendments suitable to use in apple orchards, obtained from local biomasses from agricultural and urban sources in mountain regions, from a circular economy point of view, and (b) to study the effects on soil properties after long-term monitoring of two different amendments. 

## 2. Materials and Methods

### 2.1. Soil Improvers and Their Characterization

The matured manure (MM) has been obtained after a fast and simple aeration process at the farm level, located in the Non-Valley (Trentino Province, Italy). The straw previously used as litter for animal housing at the recommended dose of about 4 kg/head*day, was added as a bulking agent to guarantee good aeration and C/N balance of the pile. The manure has been stored directly in a pile for at least three months, then covered with sheets of geotextile. The adequate provision of oxygen for the process has been guaranteed by at least 7 turnings during the storage time, by using a tractor-pulled compost turner.

The compost from digestate (CO) has been provided from a full-scale AD plant, located near Trento (Bioenergia Trentino–BET Trentino Province, Italy), which treats about 40,000 tons of OFMSW and 14,000 tons of green waste, source separately collected on all the provincial territory. The AD digestate was produced using Kompogas technology, which permits the double exploitation of organic fraction of municipal solid waste and lignocellulosic waste and residues. The thermophilic process is carried out for 21 days at 51 °C in two horizontal mixed continuously stirred horizontal tank reactors (CSTR) fed continuously. One part of the digestate is anaerobically recirculated as inoculum, the other part is sent to the subsequent composting phase after addition with yard waste to balance the C/N ratio of the starting mixture. The mix ratio for digestate composting was 50% digestate, 33% screened waste, and 17% green waste. The plant produces about 14,000 Mg/y of CO. The composting plant has eight aerated biocells and a followed maturation section in forced aerated piles. The aerobic treatment lasts about 20 days. The flowchart of amendments productions is provided in Supplementary Materials (Figure S1).

The soil improvers have been characterized regarding pH, EC, TS, and VS according to standard methods UNI (1998) [21] NTK, NNH_4_, P, and total organic carbon (TOC) according to APHA methods (2005) [22]. The dynamic respiration index (DRI, mgO_2_/kg_VS_*h) was determined with an AIR-NL dynamic respirometer using 1 kg of the sample following the aerobic process. The final value of DRI has been expressed as a 24-hour moving mean. The piles of digestate from OFMSW composting were sampled for biological stability evolution assessment (DRI measurement) at the beginning of the aerobic phase, after the intensive phase (exit from the biocells, after about 7 days), and at the end of the process (after 20 days). The piles of manure maturation were sampled at the beginning of the aerobic process, after 42 days, and at the end of the process (85 days).

### 2.2. Experimental Design

Long-term monitoring has been carried out in three different apple orchards chosen by different soil tillage, located in the Non-Valley (Trentino Province, Italy). The soils were labeled as D1, D2, and N1. The amendments were applied in the Spring of 2015 before flowering at different doses, 20 Mg/ha for CO and 40 Mg/ha for MM. Two orchards were replanted orchards (D1 and D2), while the third one was a productive apple orchard (5 years) (N1). All of them cultivated the Red Delicious apple variety, grafted on the M9 rootstock. D1 is located 330 m above sea level and was a replanted orchard. Before planting, in Autumn, the soil of D1 has undergone deep work with the removal of the fertile layer, which was again placed above the fill material after the processing. D2 is located at the same altitude as D1 and was a replanted orchard. The soil was the same as D1. Before planting, in Spring, the soil has been subjected to light processing by mechanical digging of the fertile layer. N1 is located 550 m above sea level and was a productive orchard. It has been subjected to important remediation work with the total removal of the fertile layer in 2011. The three experimental sites have been chosen to study the effect of amendments in different conditions (fields): in replanted orchards with different soil tillage; and in a productive orchard. The orchards have been fertilized each Spring with NPK chemical fertilizers as recommended by the local quality protocol in force (about 70–80 kg_N_/ha; 35–40 kg_P_/ha; 80–90 kg_K_/ha).

The randomized replicates (each replicate consists of an area of 7 m along the row, which includes about 9–10 plants) for each treatment in each soil were monitored. The soils were sampled in the fertile layer (about 20 cm depth) in each treatment area considered (Control, CO, and MM). Four technical replicates (subsampling) in each area were collected by using an Edelmann auger (diameter, 70 mm). The sampling for chemical analysis of amended (or not amended, for the Control) soil has been made each year for three years (2015-2016-2017) in two seasons (Spring and Autumn). The overall sampling point times were six (T0, T1, T2, T3, T4, and T5). T0 corresponds to the soil data of each treatment area, before amending. Detailed descriptions of the sampling points were provided in the Supplementary Materials (Table S1). The treatments considered were Control (area did not amend, but only fertilized with chemical NPK each year); CO (area amended with compost from digestate, CO, and fertilized with chemical NPK each year); and MM (area amended with matured manure, MM, and fertilized with chemical NPK each year).

Moreover, weather data (temperature and rainfall) from the three years of monitoring are provided In the Supplementary Materials (Table S2). Considering the slight differences among temperatures (mean T, minimum T, and maximum T, rain (mm) during the monitoring time, and the altitude of the sites, we assume that the weather conditions and altitudes could have a negligible effect on the parameters followed (Tables S1 and S2). 

### 2.3. Soils Monitoring

All soil samples were dried at room temperature and sieved <2 mm. An aliquot was then ground <0.02 mm for the analysis of organic carbon, total inorganic carbon, total N, P, and K. Soils were monitored during the entire study for the following parameters: pH in water 1:2,5, determined with an Inolab level 2 pH-meter (WTW, Weilheim, Germany), available P (Olsen-P) by the colorimetric method after sodium bicarbonate extraction using an ONDA V10 spectrophotometer (Giorgio Bormac, Modena, Italy) and following ISO 11263:1994, exchangeable K by ammonium acetate 1M pH 7 extraction and quantification with an Optima 8300 ICP-OES (Perkinelmer, MA, USA), total inorganic C by the volumetric method after acidification using a calcimeter (ISO 10693:1995), total C and N by dry combustion using a Vario Macro CN analyzer (Elementar, Langenselbold, Germany) and following ISO 10694:1995 and ISO 13878:1998 respectively. Organic C was calculated as the difference between total C and total inorganic C and then multiplied by 1.724 to obtain SOM. Particle-size analysis (by sieving and with a hydrometer), cation exchange capacity (CEC, with BaCl_2_ and TEA, pH 8.1, ISO 13536:1995), soluble B (ICP-OES quantification after MgCl_2_ extraction), available Cu, Fe, Mn and Zn (ICP-OES quantification after DTPA, CaCl_2_ and TEA extraction as reported previously [23]) were analyzed at the first sampling point (T0) only. Total P and K (ICP-OES quantification after aqua regia extraction following ISO 22036:2008 and ISO 12914:2012) were determined at T0, T1, and T2. 

### 2.4. Data Analysis

The data were analyzed by one-way ANOVA and multiple pairwise comparisons with Tukey formulation (level of significance α = 0.05). The comparisons were performed to study the effect of MM and CO compared to the Control during the three years of monitoring (from T0 to T5 of sampling points). The difference among fields and between treatments (CO vs. MM vs Control) for each orchard has been performed by multivariate chemometric PCA analysis. Inferential statistics were performed through STATISTICA 9.0 software (Statsoft Inc., Tulsa, OK, USA) and the PCA analysis and the graphs were through R-studio.

## 3. Results

### 3.1. Amendments Characterization

The amendment and fertilizing properties of the two tested products were verified by means of the chemical analysis and the characterization of the biological stability through the determination of the dynamic respirometric index (DRI).

**Figure 1:**
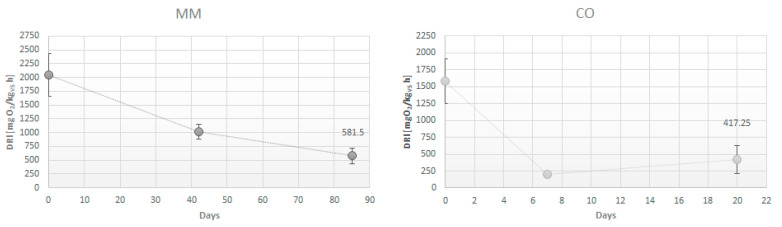
Oxygen uptake rate evolution during the maturation process (for MM) and composting (for CO).

The chemical characterization and the DRI values of the final products are reported in Table 1. The measured parameters are pH, electrical conductivity (EC), dry matter content (DM) the macronutrients, also considering the organic and mineral fraction of N, and the TOC content. Figure 1 shows the evolution of the biological stability of each product sampled during the maturation (MM) and composting (CO) processes. 

pH and EC are similar for CO and MM. CO had a higher content of DM (+29%) and for this reason, the TOC content, when expressed on fresh weight, was about 112 g/kg TOC for MM and 186 g/kg TOC for CO. The nitrogen content was different, but the organic nitrogen fraction was prevalent, while the ammonia content was negligible for both the amendments considered. The same difference was found for P and K content. The C/N ratio differs among the two products and was higher for MM. Both the products were in compliance with the limits and expected values for good quality soil improvers, reported in the Italian Law and the recent EU Regulations [8,24]. The heavy metals content has been verified on both products and the data are reported in the Supplementary Materials (Table S3). For both, the heavy metals content (Cu, Zn, Pb, Ni, Cs, Cr) was in compliance with the limits and expected values for good quality soil improvers, reported in the Italian Law and the recent EU Regulations [8,24]. Particularly the organic carbon content was greater than 20% DM or greater than 7.5% on the fresh matter, and the nitrogen had an organic nitrogen fraction exceeding 80% of the total nitrogen. The C/N ratio was less than 25 and slightly higher for MM compared to CO.

Also as regards the biological stability of the amendments the results were comparable (Table 1). The oxygen uptake was in accordance with the value expected 25 mmol/Kg_SV_ for both the products, MM and CO, highlighting the reaching of good biological stability [8]. Manure maturation lasted 90 days and after 42 days the DRI results were half of the beginning (Figure 1). OFMSW digestate composting was fast and at the end of the aerobic treatment in the biocells (after 7 days) the DRI values were similar to the final compost (Figure 1). 

The recommended doses used for apple orchard application and used in this experimental work determined similar application doses of both TOC and the main nutrients. 10.7 Mg/ha DM (+/−2.3) and 12.67 Mg/ha DM (+/−1.1) and 4.48 Mg/ha TOC and 3.73 Mg/ha TOC were applied for MM and CO respectively. The nitrogen and phosphorus contents were 0.25 Mg/ha N and 0.25 Mg/ha (MM and CO, respectively) and 0.09 Mg/ha P and 0.08 Mg/ha P (MM and CO, respectively). The K content was the main different nutrient compared to the two products, considering the doses applied: 0.12 Mg/ha K for MM and 0.05 Mg/ha K for CO.

### 3.2. Soils Monitoring: Nutrient and SOM

**Figure 2:**
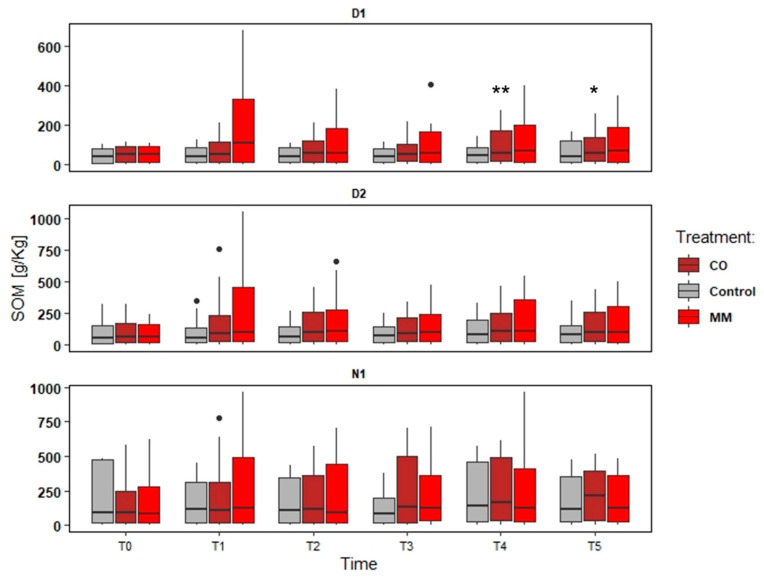
Soil organic matter (SOM) trends (T0–T5) in three experimental fields (D1, D2, and N1) comparing soil amended with compost from digestate (CO), soil amended with matured manure (MM), and soil without amendment application (Control). The significantly different means per sampling date (T0–T5) are identified through asterisks; ** = *p* > 0.01; * = *p* > 0.05; • = outliers.

**Figure 3:**
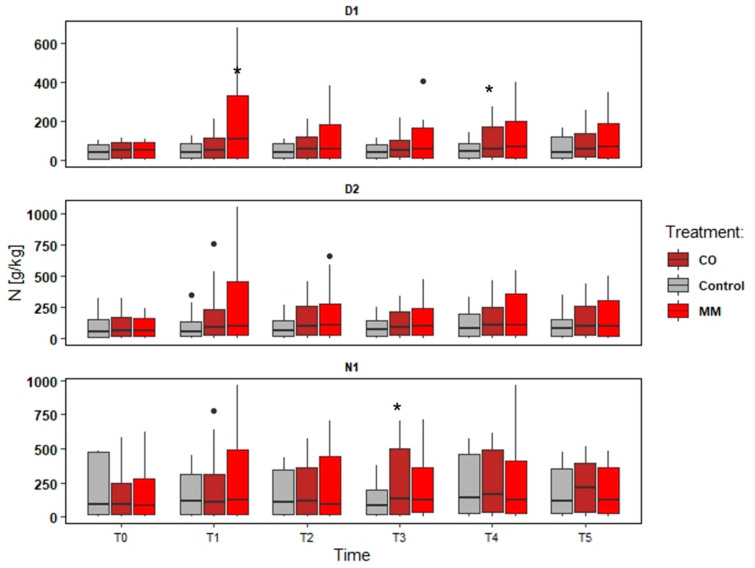
Total nitrogen content (N) trends (T0–T5) in three experimental fields (D1, D2, and N1) comparing soil amended with compost from digestate (CO), soil amended with matured manure (MM), and soil without amendment application (Control). The significantly different means per sampling date (T0–T5) are identified through asterisks; * = *p* > 0.05; • = outliers.

**Figure 4:**
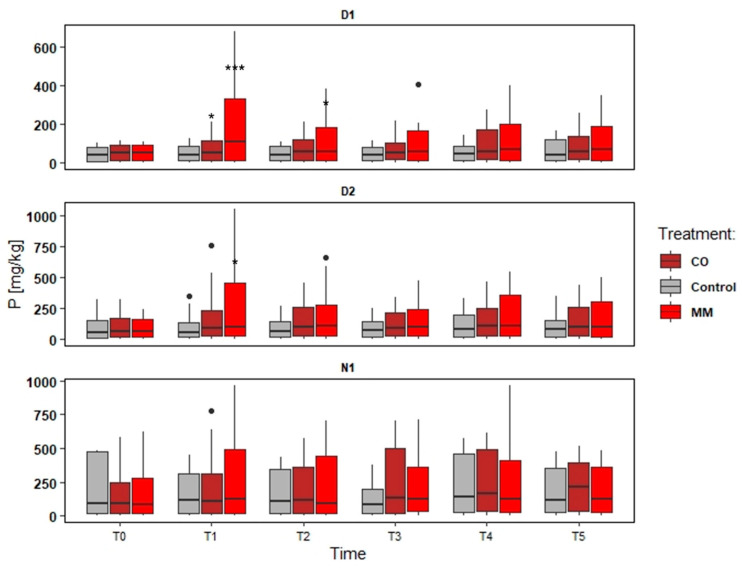
Phosphorus content (available P) trends (T0–T5) in three experimental fields (D1, D2, and N1) comparing soil amended with compost from digestate (CO), soil amended with matured manure (MM), and soil without amendment application (Control). The significantly different means per sampling date (T0–T5) are identified through asterisks; *** = *p* > 0.001; * = *p* > 0.05; • = outliers.

**Figure 5:**
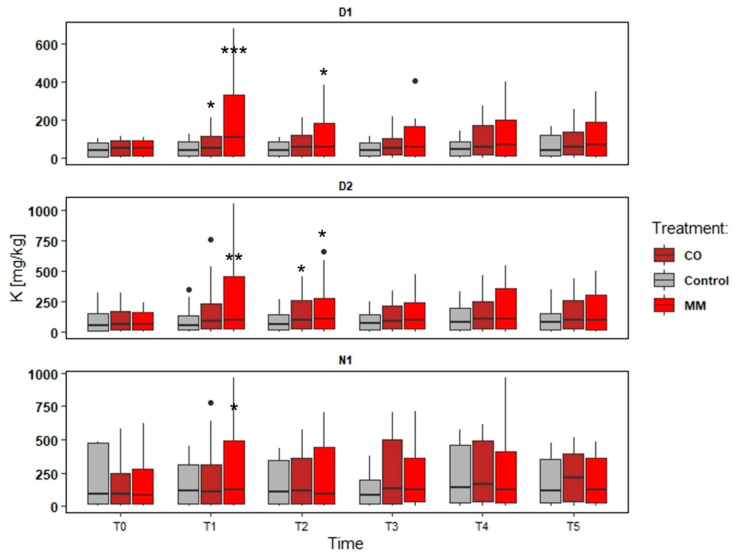
Potassium content (available K) trends (T0–T5) in three experimental fields (D1, D2, and N1) comparing soil amended with compost from digestate (CO), soil amended with matured manure (MM), and soil without amendment application (Control). The significantly different means per sampling date (T0–T5) are identified through asterisks; *** = *p* > 0.001; ** = *p* > 0.01; * = *p* > 0.05; • = outliers.

The soil characterizations are reported in Table 2 and the following figures (Figure 2, Figure 3, Figure 4 and Figure 5). Table 2 reports the preliminary characterization, CEC, and micronutrients of the soil from the different apple orchards at the beginning of the monitoring. The figures and multivariate analysis (PCA) consider the organic matter (SOM) and macronutrients (N, P, K, and Mg) in the soil after two amendments application (CO, soil amended with compost from digestate, and MM, soil amended with matured manure) compared to the Control (area without amendments application), during three years of monitoring (from T0 to T5), in the three fields (D1, D2, and N1). 

The preliminary characterization of the apple orchards showed similar conditions as regards the texture, pH, CEC, and micronutrients (Table 2). 

The data elaboration aims to understand both the effect of different amendments and different fields, also considering different soil management (tillage). The data collected were provided in the following Figures (Figure 2, Figure 3, Figure 4 and Figure 5).

The increase in the nutrient content was more evident in D1, as well as SOM. The tendency to increase was observed also in N1, while in D2 the main difference was in the first years, comparing the first sampling point (Spring) and the second one (Autumn). In D1, the MM application determined a slight increase of N and SOM content (about +33% N and +50% SOM) (Figure 2 and Figure 3), but a significant increase of P and K (about +180% P and +438% K) compared to Spring and Autumn conditions (T0 and T1, in the first monitoring year), significantly higher of the Control increase too (Figure 4 and Figure 5). At the end of the orchards monitoring (T5) the N and SOM content was similar to the value registered in the autumn of the first year and similar to the Control data, while the P and K content decreased (about −42% P and –51% K) (Figure 2, Figure 3, Figure 4 and Figure 5).

In D2, the MM application determined a less increase compared to D1 data (+9% N, +22% SOM comparing T0 and T5) and confirmed the increase of P and K content (about + 11% P and + 164% K) in the first monitoring year (T0 and T1) (Figure 3). N1 registered greater (but not significant) effects for N and SOM (T0 vs. T5) (+34% and +42%) than Control, but the effect on P and K content was negligible at all the sampling points (Figure 2, Figure 3, Figure 4 and Figure 5). 

The CO application determined different conditions. In all the fields the effect on P and K was negligible, also considering the increase of the Control, while the effect on N and SOM was greater considering the comparison to T0 and T5 for D1 (+37% N and +83% SOM) (Figure 2) and N1 (+77% N and +94% SOM) (Figure 2, Figure 3, Figure 4 and Figure 5), even if not significant compared to the Control trend. D2 differed and the increase of N and SOM was less compared to other fields (+13% N and +41% SOM) (Figure 2 and Figure 3). However, the increase of SOM and N was detected also in the Control, which was not significantly different from MM data (Figure 2 and Figure 3). 

**Figure 6:**
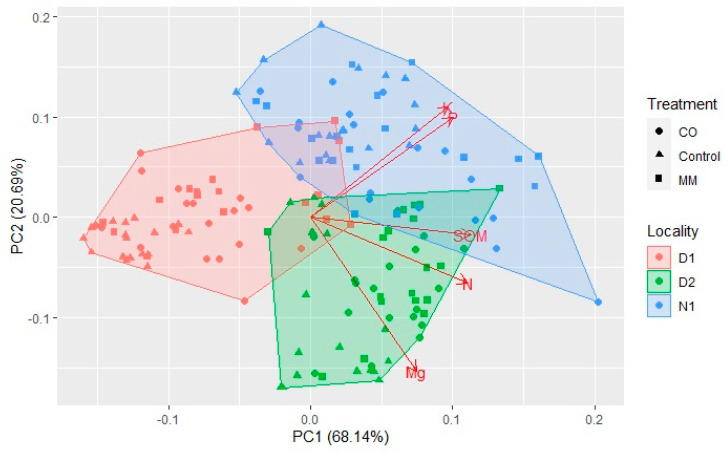
Multiparametric differences among Locality (D1, D2, and N1) and among Treatments (Control, CO, and MM) according to PCA analysis. The different colors identified the Locality, and the different shapes identify the Treatment within each experimental site. The biplot showed the correlations among the variables (SOM, N, Mg, P, and K).

The PCA analysis of the data obtained after three years of monitoring is reported in Figure 6 and was performed considering the chemical parameters measured to understand the overall effect on soils considering fields and treatments. 

The principal component 1 (PC1) explains 68.14% of the variance, while PC2 explains 20.69% of the variance, all explaining 88.83% of the total variability. The PC1 clusters clearly D1 from D2 and N1. The D1 showed a very different response to the treatments compared to other fields; the amending effect is greater. In this regard, it is important to consider the starting conditions of the three fields, which are different. Before the amendment application, the SOM content of D1 (12 g/kg) was significantly lower than others (31.3 g/kg for N1, 28 g/kg of D2), as well as the N content (0.9 g/kg D1, 2.0 g/kg D2, and 1.6 g/kg N1). The same difference was found for P and K content; D1 (70.33 g/kg P, and 101.66 g/kg K) differed for the starting conditions from D2 (124.33 g/kg P, and 22.66 g/kg K) and N1 (359.66 g/kg P, and 481.33 g/Kg K). The vertical axis clusters D2 from N1. The correlations among the variables (biplot) showed a positive correlation in PC1 for all the variables considered, while PC2 showed correlations with P and K.

### 3.3. Phosphorus and Potassium Dynamics

**Figure 7:**
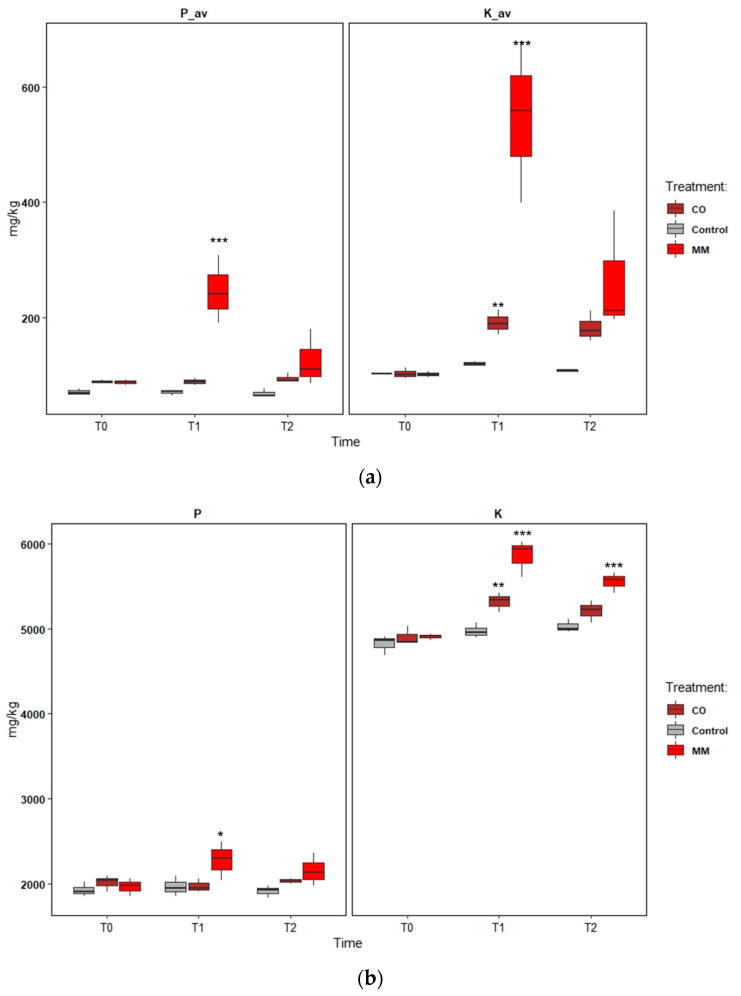
(**a**) Available P (P_av) and exchangeable K (K_av) and (**b**) total P (P) and total K (K) fractions dynamics in the first year after amendment application (from T0 to T2) in one experimental field (D1), comparing soil amended with compost from digestate (CO), soil amended with matured manure (MM), and soil without amendment application (Control). The significantly different means per sampling date (T0–T2) are identified through asterisks; *** = *p* > 0.001; ** = *p* > 0.01; * = *p* > 0.05.

The data of the available P and K fractions and total P and K fractions are reported in Figure 7a,b respectively.

The analysis of these two fractions has been performed for a better understanding of the strong increase of available P (P-Olsen) fraction and K exchangeable in the MM treatment areas. The MM application determined a significant increase of available P (+180%, from T0 to T1) and more lightly total P (+16%). The next year the available P decreased (−49%, from T1 to T2), and total P decreased slightly (−5%). The same trend showed the K fractions. Exchangeable K and total K increased in the first year (+438%; +19%, from T0 to T1), while in the next year, exchangeable K significantly decreased (−51%, from T1 to T2), and total K too (−5%, from T1 to T2) (Figure 7a,b). CO application led to an increase in exchangeable K (+86%, from T0 to T1) and this increase was detected also in total K (+9%). The effect on both the P fractions of CO application was lower (Figure 7a,b).

## 4. Discussion

The work aims to study both the quality of different kinds of soil improvers available locally for fruit-growing sectors and the specific effects on soil. The matured manure and compost from digestate application have been made in the apple orchard with the provision of recommended NPK mineral fertilizers for apple growing too. Normally, the farmers use alternatively MM and CO for SOM management in their orchards. The study of the amendment and fertilizers properties of the products could provide information to reduce the mineral fertilizers use and at the same take care and improve the management of the soil quality and biodiversity in intensive agriculture.

### 4.1. Amendment and Fertilizers Properties of Soil Improvers Used

Compost from digestate and mature manure are two products available locally in rural areas. Both products may use for SOM management. The chemical characterization showed good environmental and agronomic quality, and both are suitable for orchard application. The MM has less content of DM, but a greater content of TOC, P, and K compared to CO. N content is similar for MM and CO, as well as the biological stability determined after DRI measurement, showing the aerobic transformation of the OM and mineral nutrients. The compliance of all the parameters, including heavy metals contents with Italian Law and EU Regulations has been verified [8,24]. The analysis of biological stability during aerobic treatment of the OFMSW digestate confirms the results of previous works [25,26].

The intensive livestock sectors and the use of raw manure directly on soil could have a depleting effect on the environment considering the increase of GHG, soil, and water pollution by leaching nutrients and phytotoxic elements. The concentration of many animals in a restricted area requires the introduction of treatments, which permit manure delocalizing [27,28,29]. The fast and controlled maturation of the manure led to a product with reduced water content and high content of stabilized OM and nutrients, suitable for application in the apple orchard. At the same time, the process is less difficult than composting and may be managed at the farm level [28,30]. The greater cost of manure composting is due to transport and distribution; the creation of a small supply chain and the production of a user-friendly product (more DM content) can contribute to wider use in other agricultural sectors (such as orchards/vineyards) [10,31,32]. As previously detected the composting enhances P available, while leading to a C and N reduction, compared to raw manure (data of this work does not show) [26,33]. 

The digestate obtained from OFMSW anaerobic digestion contains mineral nitrogen, and microelements could have a role as fertilizers for plant growth. Its composting allows for overcoming the issues regards the health risks for animals and humans, removing the presence of phytotoxic biological compounds and improving its handling [25]. The treatment of OFMSW through anaerobic digestion, prior to composting, has a more beneficial effect on the environment than direct composting of the OFMSW [34]. The compost from digestate have generally a less content of organic C and total N compared to compost from raw feedstock, and from manure too [34]. The C/N ratio of CO was less than MM considering the TOC value, which is half compared to MM. The digestate has a very low C/N ratio; the AD process maintains the nutrient content of the feedstock and the nitrogen content ranges from 1.1% to 9.6%, higher than the fresh OFMSW [17]. The composting of digestate requires the proper adjustment of the C/N ratio by the addition of lignocellulosic waste which has the function of bulking agent, which also increases the aromatic-C in the final compost [19]. Moreover, the organic matter fraction of digestate contains a more recalcitrant fraction of OM from the OFMSW, due to the mineralization occurring during the anaerobic process, which leads to a higher fraction of easily degradable compounds consumption [35,36]. For both the previous reasons, the compost from digestate may be considered different from the compost from raw OFMSW. 

The considerations regarding the quality and the properties of the soil improvers are a preliminary step and the study of the effect on soil and plant is crucial for better identifying the proper use. In general, the use of matured and stabilized products avoids the environmental issue occurring due to the use of fresh products [37]. Moreover, other factors, such as the good infrastructure for collection, handling, storage, distribution, and sanitation are important to promote a wider farmer’s use [38]. 

### 4.2. Effect on the Soil of Compost from Digestate and Mature Manure

CO and MM applications determine different effects, even though both are derived from an aerobic process. The characterization data (Table 1) and the chosen doses led to similar doses of TOC, N, and P. Only the K differed between CO and MM applications. The data and PCA analysis reported in this work demonstrated a good correlation between SOM and N, and between P and K. The MM determined a greater effect on P and K, while the main effect of CO application was in SOM and N increase. The analysis of P and K in both the fractions, total and available, confirmed the contribution of MM on the P and K available for the plant increase, while the CO application effect was negligible. 

The different contributions of SOM in soils confirm the importance not only of the quantity but also of the quality of OM applied, as detected in long-term monitoring of soils amended with products with low C/N [39]. Soil organic amendments can enhance soil C storage through multiple mechanisms; these include increasing soil microbial biomass and activity, enhancing soil water-stable aggregation, and introducing recalcitrant C [39,40]. The application of CO generates a positive impact on C and as suggested previously, the long-term CO application maybe promotes the use of a different source of C from the microorganism determining an enlarging soil microbial available C pool, higher soil microbial biomass, and increasing aggregate formation [41]. Moreover, the soil improvers with low C/N seem to contribute to enhancing SOM due to their slow decomposition [39]. Conversely, the MM application does not show a significant increase compared to the Control suggesting a different impact on SOM of native soil. We hypothesize that the higher content of the most degradable C improved SOM decomposition and nutrient mineralization [39]. Microbial P and N uptake can prevent P fixation by soil colloids or N leaching. When the readily available organic carbon is depleted, microbial biomass N and P become available to plants through microbial turnover. Microorganisms can thus be conceptualized as slow-release fertilizers that store P and other nutrients when their concentrations are high, and then release them when nutrient concentrations are low [39]. The data of P availability confirm the previous explanation, considering the strong increase in the first year of application, which exceeds the dose applied with MM. The P availability depends on microbial activity; the addition of organic products stimulates the extracellular enzymes enhancing organic matter decomposition and P mineralization [42,43]. P is released slowly over time as the concentration of easily available C decreases [43]. The difference in K availability seems to be more linked to the dose applied and due to the same pathways. Moreover, the temporary difference between CO and MM applications provides useful information about their frequency of use (every three years or every two years).

### 4.3. Differences between Apple Orchards on Soil Improvers Effects

Significant differences were found also in comparing the orchards monitored. The D1 and D2 were both replant apple orchards; D1 has been subjected to deep work with the removal of the fertile layer, D2, instead has been subjected to more light processing by mechanical digging of the fertile layer, and N1 was a 5-year productive orchard and has been subjected to a remediation work with the total removal of the fertile layer, before planting. The different processing of the soil could explain the different starting conditions, particularly as regards the SOM and N content. The texture analysis demonstrated the very similarity of the soil, and the difference does not due to the physical conditions. Based on previous work, the accumulation of nutrients was greater in silt sites than in sandy sites, where the leaching of organic compounds may have occurred [37]. Moreover, it has been found that fine-textured soils (silty loam and silty clay) had the highest CO_2_ emission, and the manure rate had a great impact on GHG emissions [37,38]. Probably the starting condition of the fields significantly affected the SOM and nutrient dynamics after amendment. Proper soil tillage has a strong effect on soil response to amending.

The work demonstrated the crucial role of the specific knowledge of soil and soil improvers used to obtain the expected fertilizing and amendment effects. The data on P and K dynamics pose a crucial question regarding the possibility of drastically reducing phosphate and potassium fertilization in apple orchards with proper management of SOM. 

## 5. Conclusions

The work aims to study the properties of different kinds of soil improvers available locally for fruit-growing sectors. Normally, the farmers use alternatively MM and CO for SOM management in their orchards, but the data collected showed different effects of the two products. Both, MM and CO have characteristics compatible with high-quality amendments, and the aerobic process (fast maturation of manure and OFMSW digestate composting) determined the increase of biological stability and reduction of mineral nutrients. The study of the amendment and fertilizing properties of the products provide useful information to limit the use of mineral fertilizers and at the same time to take care and improve the management of the soil (particularly as regards SOM) in intensive agriculture. 

Moreover, the work demonstrated the crucial role of the specific knowledge of soil and soil improvers used to obtain the expected fertilizing and amendment effects. The work reports data about the long-term monitoring of two different amendments, highlighting the relevance of different factors on nutrients and SOM, such as soil tillage, soil management, and the kind of soil improvers used. The work suggests interesting future studies on the fertilizing effect, to reduce mineral fertilization. The data on P and K dynamics pose a crucial question regarding the possibility of drastically reducing phosphate and potassium fertilization in apple orchards with proper management of SOM. In line with the circular economy strategy, the exploitation of organic matter–related nutrients available at the local level promotes the reuse of bioresources, the reduction of fossil fertilizers, and the implementation of sustainable and efficient synergies between the livestock sector and urban-waste recovery systems with other agricultural sectors.

## Figures and Tables

**Table 1:** Amendments characterization. The significantly different means are identified through asterisks (* = *p* > 0.05).

Amendments	pH	EC	DM	TOC	NTK	NNH_4_	P	K	C/N	DRI
	--	mS/cm^2^	%	%DM	%DM	mg/kg	%DM	%DM	--	mgO_2_/ kg_VS_*h
MM	8.81 ± 0.32	2.51 ± 0.42	26.82 ± 3.97	41.8 ± 8.0	2.36 ± 0.25	386 ± 193	0.90 ± 0.31	2.82 ± 2.05	17.71	571.75 ± 170.03
CO	8.77 ± 0.23	2.77 ± 0.32	63.38 ± 3.80	29.4 ± 7.2 *	2.05 ± 0.35 *	284 ± 200	0.65 ± 0.36 *	1.30 ± 0.43 *	14.54	417.25 ± 182.1

**Table 2:** Soil preliminary characterization. Micronutrient, pH, CEC, and texture data of each treatment before amending (T0); comparing soil from the area before amending with compost from digestate (CO), soil from the area before amending with matured manure (MM), and soil from the area without amendments application (Control).

Apple Orchard/ Treatment Area	Sand	Silt	Clay	pH	CEC	B	Fe	Mn	Cu	Zn
	%	%	%	--	meq/100 g	mg/kg	mg/kg	mg/kg	mg/kg	mg/kg
D1										
Control	58.6	18.4	23	7.7	12.37	0.39	21.03	11.37	11.83	2.87
CO	56.1	20.9	23	7.8	12.83	0.37	20.33	11	10.67	2.87
MM	55.6	24.4	20	7.7	12.50	0.44	20.93	11.37	11.03	2.83
D2										
Control	57.7	25.3	17	6.7	16.13	1.14	64.60	37.53	19.27	10.63
CO	56	29	15	6.8	17.03	1.17	52.37	18.87	18.4	13.57
MM	57	28	15	7	16.83	1.19	57.63	44.57	18.90	10.87
N1										
Control	60.40	26.93	12.67	7.63	14.33	0.76	16.23	15.53	14.17	5.87
CO	60.83	25.83	13.33	7.7	15.57	0.66	14.50	6.50	12.20	5.10
MM	61.90	26.10	12	7.67	13.23	0.7	14.9	9.93	13.57	5.33

## Data Availability

Not applicable.

## Supplementary Materials

The following supporting information can be downloaded at: https://www.mdpi.com/article/10.3390/ijerph192315512/s1, Figure S1: Flow chart of the production of the two amendments used in this work. The first flowchart regards the composting of organic fraction of municipal solid waste digestate and the second one the aerobic treatment (maturation) of manure; Table S1: Sampling points: time and description; Table S2: Weather data for the experimental periods of monitoring. T, mean of the temperature of the month considered. T MIN, the minimum temperature registered during each month considered. T MAX, the maximum temperature registered during each month considered. Rain [mm], the sum of the rainfall for each month considered; Table S3: Heavy metals content of the two products used, compost from digestate (CO) and matured manure (MM).
