# Supplementary material for: Matured Manure and Compost from the Organic Fraction of Solid Waste Digestate Application in Intensive Apple Orchards
Source: Int J Environ Res Public Health. 2022 Nov 23;19(23):15512. doi: 10.3390/ijerph192315512 (PMC9739789; doi:10.3390/ijerph192315512)
Supplement: Supplementary file 1 [file ijerph-19-15512-s001.zip › ijerph-2004682-supplementary.pdf]

International Journal of Environmental Research and Public Health

MDPI

Article

Matured manure and compost from the organic fraction of solid waste digestate application in intensive apple orchards

Daniela Bona[1*], Andrea Cristoforetti[1], Roberto Zanzotti[1], Daniela Bertoldi[1], Nicole Dellai[1], Silvia Silvestri[1]
[1] Fondazione Edmund Mach, Via E. Mach, 1, 38010 San Michele a/A, Italy
* Correspondence: daniela.bona@fmach.it

Supplementary Materials

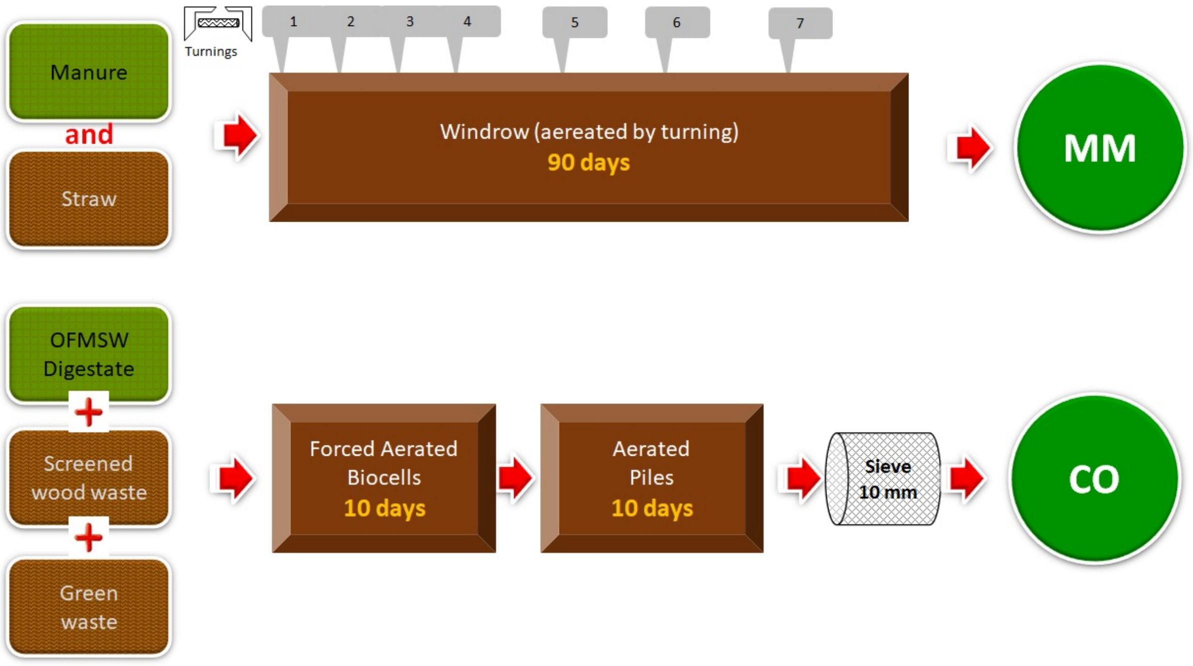

Figure S1. Flow chart of the production of the two amendments used in this work. The first flowchart regards the composting of organic fraction of municipal solid waste digestate and the second one the aerobic treatment (maturation) of manure.

Table S1. Sampling points: time and description.

Sampling points	Time	Description
**TO**	April 2015	Phenological apple phase: Bloom
**T1**	October 2015	Phenological apple phase: post-harvesting
**T2**	June 2016	Phenological apple phase: growing fruit or “fruit-nut” phase
**T3**	October 2016	Phenological apple phase: post-harvesting
**T4**	June 2017	Phenological apple phase: growing fruit or “fruit-nut” phase
**T5**	October 2017	Phenological apple phase: post-harvesting

International Journal of Environmental Research and Public Health

MDPI

Table S2. Weather data for the experimental periods of monitoring. T, mean of the temperature of the month considered. T MIN, the minimum temperature registered during each month considered. T MAX, the maximum temperature registered during each month considered. Rain [mm], the sum of the rainfall for each month considered.

Year	Month	D1 and D2				N1			
		T [°C]	T MIN [°C]	T MAX [°C]	Rain [mm]	T [°C]	T MIN [°C]	T MAX [°C]	Rain [mm]
2015	04	12,6	4,4	20,4	15,4	12,3	5,8	18,8	13
2015	05	16,8	10,4	23,2	115,2	16,3	11	22	107,8
2015	06	20,2	13,3	27,3	98	19,7	14,1	26,4	117,4
2015	07	25	17	32,8	18,8	24,3	18,3	31,3	24,8
2015	08	22,5	15,8	30,1	66	22	16,3	28,7	61,6
2015	09	15,7	10,3	22,6	160	15,5	10,9	21,4	124,2
2015	10	10,9	7,2	17,2	152,8	10,7	7,2	16	143,8
2015	11	4,8	0,1	13,8	1	5,8	1,3	13	0,6
2015	12	0	-3,7	7,7	0	1,8	-2	8,7	0
2016	01	-0,4	-4,3	6,4	30,8	0,6	-3	5,9	23,2
2016	02	3,8	-0,2	9,5	125,2	3,9	0,4	8,2	125,8
2016	03	7,6	1,5	14,5	75,4	7,3	2,5	13,1	75,4
2016	04	13,2	6,9	19,7	79	12,5	7,3	18,4	35,8
2016	05	14,6	8	20,7	137,4	14,3	9	19,8	134,2
2016	06	19,4	13,6	25,7	113,6	18,9	13,8	24,9	83,6
2016	07	21,8	15,5	28,7	96,2	21,5	16,3	27,8	96,8
2016	08	20,4	14,1	27,7	70,2	20,3	14,8	26,9	84,6
2016	09	18,7	12,4	27	22,8	18,5	13,4	25,3	20,6
2016	10	10,6	5,9	17,5	68,2	10,4	6,5	15,8	55,2
2016	11	5,3	1,9	10,8	104,8	5,3	2,4	9,5	85,8
2016	12	-0,2	-4,3	8,8	2,6	1,5	-2,6	8,9	0
2017	01	-2,7	-8,1	5,9	3	-1,6	-6,2	4,4	1,6
2017	02	4,1	-0,3	10,7	84	4,3	0,8	9,3	76,8
2017	03	10	2,7	18,5	27,6	10	4,2	16,8	27,6
2017	04	11,9	4,3	19,8	61,6	11,7	5,8	18,1	47,6
2017	05	16,8	9,6	24,3	76,6	16,1	10,4	22,6	74,4
2017	06	21,6	14,1	28,8	113,2	21,1	15,1	27,4	129,8
2017	07	22	14,3	29,6	82,6	21,5	15,1	28,1	67,6
2017	08	21,7	14,6	29,9	194,2	21,4	15,3	28,4	218,2
2017	09	14,9	10	21,3	125,8	14,3	10	19,7	145,2
2017	10	10,5	4,4	19,9	10	11,4	6,1	18,4	12,8

International Journal of Environmental Research and Public Health

MDPI

Table S3. Heavy metals content of the two products used, compost from digestate (CO) and matured manure (MM).

Amendments	Cu	Zn	Ni	Pb	Cd	Cr
	mg/kg	mg/kg	mg/kg	mg/kg	mg/kg	mg/kg
MM	49.77	182.77	5.61	2.17	<1	--
CO	97	168.75	16.13	31.65	0.75	<0.05
